# Mapping a double flower phenotype-associated gene *DcAP2L* in *Dianthus chinensis*

**DOI:** 10.1093/jxb/erz558

**Published:** 2020-01-28

**Authors:** Qijian Wang, Xiaoni Zhang, Shengnan Lin, Shaozong Yang, Xiuli Yan, Mohammed Bendahmane, Manzhu Bao, Xiaopeng Fu

**Affiliations:** 1 Key Laboratory of Horticultural Plant Biology, College of Horticulture and Forestry Sciences, Huazhong Agricultural University, Wuhan, China; 2 Key Laboratory of Urban Agriculture in Central China (pilot run), Ministry of Agriculture, Wuhan, China; 3 Laboratoire Reproduction et Development des Plantes, INRA-CNRS-Lyon1-ENS, Ecole Normale Supérieure de Lyon, Lyon, France; 4 Fondazione Edmund Mach, Italy

**Keywords:** BSR-seq, *APETALA2 (DcAP2L)*, ddRAD, *Dianthus chinensis*, double flower trait, miR172, QTL mapping

## Abstract

The double flower is a highly important breeding trait that affects the ornamental value in many flowering plants. To get a better understanding of the genetic mechanism of double flower formation in *Dianthus chinensis*, we have constructed a high-density genetic map using 140 F_2_ progenies derived from a cross between a single flower genotype and a double flower genotype. The linkage map was constructed using double-digest restriction site-associated DNA sequencing (ddRAD-seq) with 2353 single nucleotide polymorphisms (SNPs). Quantitative trait locus (QTL) mapping analysis was conducted for 12 horticultural traits, and major QTLs were identified for nine of the 12 traits. Among them, two major QTLs accounted for 20.7% and 78.1% of the total petal number variation, respectively. Bulked segregant RNA-seq (BSR-seq) was performed to search accurately for candidate genes associated with the double flower trait. Integrative analysis of QTL mapping and BSR-seq analysis using the reference genome of *Dianthus caryophyllus* suggested that an SNP mutation in the miR172 cleavage site of the A-class flower organ identity gene *APETALA2* (*DcAP2L*) is responsible for double flower formation in *Dianthus* through regulating the expression of *DcAG* genes.

## Teaser

An SNP mutation in the miR172 target site of the *DcAP2L* gene is associated with a double flower phenotype in *Dianthus chinensis*.

## Introduction

Genetic linkage maps play important roles in genetic and genomic studies, and provide an essential foundation for quantitative trait locus (QTL) mapping and map-based cloning. Bernatzky and Tanksley (1986) constructed the first linkage map for tomato which contains 84 restriction fragment length polymorphisms (RFLPs). Subsequently, genetic maps were developed and used in a wide range of ornamental plant species, such as petunia (Strommer *et al.*, 2000), rose (Debener and Mattiesch, 1999), azalea (Keyser *et al*., 2010), and chrysanthemum (Zhang *et al.*, 2010). Single nucleotide polymorphisms (SNPs) became the main genetic markers to construct high-resolution linkage maps after the emergence of next-generation sequencing (NGS). Restriction site-associated sequencing (RAD-seq) is widely used for genotyping and polymorphism identification (Davey and Blaxter, 2011). Subsequently, several related methods for library construction have been developed, such as 2b-RAD (Wang *et al.*, 2012) and double-digest RAD-seq (ddRAD-seq; Peterson *et al.*, 2012). The ddRAD-seq method, first published in 2012 (Peterson *et al.*, 2012), was rapidly adopted by researchers to construct high-density genetic linkage maps for QTL mapping, genome assembly, or phylogenetic analysis. It has been successfully used in diverse organisms, such as cultivated strawberry (*Fragaria*×*ananassa* Duch.) (Davik *et al.*, 2015), Japanese eel (*Anguilla japonica*) (Kai *et al.*, 2014), *Brassica napus* (Wu *et al.*, 2016), *Sebastes* rockfish (Fowler and Buonaccorsi, 2016), and two avian genera (DaCosta and Sorenson, 2016). BSR-seq, that combines bulked segregant analysis (BSA) and RNA-seq, provides a good mapping strategy to identify candidate genes and genetic markers linked to target genes (Sanzhen *et al.*, 2012). The BSR-seq approach permits identification of the *glossy13* gene associated with plant surfaces covered by epicuticular waxes in maize, and mapping of the leaf senescence gene *els1* in common wheat (Li *et al.*, 2013).

Many *Dianthus* species, such as *D. chinensis*, *D. caryophyllus* L., and *D. barbatus* L., have high economic and cultural value worldwide. *Dianthus chinensis* is a perennial herbaceous plant that has been grown as an ornamental garden plants in China and across other temperate zones for a long time (Casanova *et al.*, 2004). *Dianthus chinensis* plants are also widely used in landscaping projects, thereby encouraging breeders to focus their attentions towards producing novel varieties with high-value ornamental traits. However, the breeding work of this species remains at the level of simple cross-pollination and phenotypic selection. Only a little information is available on the molecular mechanisms associated with key ornamental traits in *Dianthus* spp.

The double flower trait is highly valued in many species. Double flowers refer to a characteristic where the number of petals per flower is double or more than the number of petals in the wild-type simple flower species. Due to its ornamental value, the double flower phenotype is selected by breeders in many species. It is considered to be one of the most cherished traits for many species such as roses, lilies, *D. caryophyllus*, etc. In the past, many studies have tackled the underlying molecular mechanisms associated with double flower formation. This started by studying putative changes in the expression and function of the floral organ identity genes of the well-established ABCE model (Irish and Litt, 2005; Krizek and Fletcher, 2005). With the exception of the class A gene *APETALA2* (*AP2*), most floral organ identity genes encode MADS-box transcription factors. *AP2* belongs to the *AP2* family classified within the *AP2/ERF* superfamily (Nakano *et al.*, 2006), and has a conserved miR172 target site (Kim *et al.*, 2006). *AP2* plays an important role in specifying sepal and petal organ identity, and in repressing C-function during flower development in *Arabidopsis thaliana* (Bowman *et al.*, 1991; Drews *et al.*, 1991). In Arabidopsis, plants with mutation of the miR172 target site in *AP2* exhibit an enlarged floral meristem and an excess of stamens, indicating an important role for *AP2* in the control of floral meristem termination (Chen, 2004).

With the exception of *D. caryophyllus*, the majority of wild *Dianthus* species have the simple flower type. Simple flower cultivars have five petals, while the number of petals in the double flower cultivars can vary from 20 to >40 petals per flower. Previously, the *D*_*85*_ locus that controls flower type in *D. caryophyllus* was mapped to linkage group (LG) 85P_15-2 using a simple sequence repeat (SSR)-based genetic linkage map which was constructed by using 91 F_2_ progeny derived from a cross between line 85-11 (double flower) and ‘Pretty Favvare’ (single flower) (Yagi *et al.*, 2014). Two SSR markers (CES0212 and CES1982) that are tightly linked to the *D*_*85*_ locus were identified and subsequently used for breeding in the *Dianthus* genus. However, no QTL mapping or candidate gene identification for this important trait have been achieved to date. The diploid *D. chinensis* has emerged as a model species for the *Dianthus* genus (Fu *et al.*, 2008), compared with *D. caryophyllus* commercial cultivars, such as *D. caryophyllus* cvs ‘Liberty’, ‘Odino’, and ‘incas’, whose genomes are tetraploid.

Here, we address the molecular mechanisms controlling the double flower trait in *D. chinensis*. A high-density linkage map was constructed through the application of ddRAD-seq. QTL mapping of the flower type phenotype and other horticultural traits was conducted, and candidate genes were selected by integrative analysis of QTL mapping and BSR-seq. This map helps the identification of *DcAP2L* as a candidate gene associated with the double flower type. We report that an SNP in the miR172 target site of *DcAP2L* is likely to be associated with double flower formation in *D. chinensis*. Moreover, the genetic linkage map we developed in this study will be instrumental for further applications in QTL-based fine mapping of other important traits.

## Materials and methods

### Plant materials and DNA isolation

The F_2_ mapping population in *D. chinensis* of 400 individuals was generated by an intraspecific cross between the single-flowered line ‘MH’ and the double-flowered line ‘X4’. ‘MH’ and ‘X4’ cultivars originated from Northern China with continuous self-crosses over 10 generations, and no trait is segregated. Data of agronomic traits [double flower trait (DFT), leaf with wax (LWW), leaf width (LW), leaf length (LL), stamen number (SN), plant height (PH), plant width (PW), major stem diameter (SD), branch number (BN), stem color (SC), calyx color (CC), and style color of the pistil organ (PC)] were measured during the full blooming stage in 2016 for the parents and for the 400 F_2_ progenies. Evaluation criteria for agronomic traits and assignment for qualitative characters are shown in Supplementary Table S1 at *JXB* online. The two parental lines and 140 F_2_ individuals with similar growth potential were selected for genotyping and mapping. Young leaf tissues from the two parents and from F_2_ individuals were harvested. Genomic DNA was then extracted using the cetyltrimethylammonium bromide (CTAB) method mainly as previously described (Doyle, 1990).

### Scanning electron microscopy

The samples for SEM were collected from flower buds at the development stage where the primordia of all floral organs are formed. Samples were fixed in a solution containing 2.5% glutaraldehyde in phosphate buffer (pH 7.0) for 24 h, and then analyzed using a JSM-6390 LV (Hitachi, Tokyo, Japan) electron microscope (microscopy platform of Huazhong Agricultural University) (Wetzstein *et al.*, 2011). SEM imaging was used to observe the number variation trend of calyxes, petals, stamens, and pistils in two parents. ImageJ was used to analyze the area change trend of the floral primordium.

### Preparation and sequencing of the ddRAD-seq library

The ddRAD libraries were constructed according to a protocol described by Peterson *et al.* (2012). *Eco*RI and *Nla*III (New England Biolabs, Ipswich, MA, USA; 20 U per reaction) were used for double digestion of 500 ng of DNA template from each individual in a single combined reaction for 30 min at 37 °C. Subsequently, a Qiagen MinElute Reaction Cleanup Kit (Qiagen, Valencia, CA, USA) was used to purify the fragmented samples. P1 adaptors [including a unique 4–8 bp multiplex identifier (MID)] that bound to the *Eco*RI-created restriction sites were added to fragments, together with P2 adaptors that bound to the restriction sites generated by *Nla*III. Each reaction contained a total volume of 40 μl: 500 ng of DNA, 1 μl of P1 adaptor (10 mM), 1 μl of P2 adaptor (10 mM), 1 μl of T4 ligase (1000 U ml^–1^), 4 μl of 10×T4 ligation buffer, and double-distilled water to 40 μl. The PCR program was used with the following conditions: 37 °C for 30 min, 65 °C for 10 min, followed by a gradual decrease in temperature of 1.3 °C min^–1^ until the temperature reached 20 °C. Fragments were selected by size (i.e. 400–600 bp) following electrophoresis on a 1% agarose gel. Paired-end (150 bp) sequencing of the ddRAD products from two parents and 140 F_2_ individuals was performed using an Illumina HiSeqXten sequencing platform (Illumina, Inc., San Diego, CA, USA).

### SNP discovery, and genotyping and construction of linkage maps

A filtering process was performed to get rid of the raw reads lacking sample-specific MIDs and the expected restriction enzyme motifs by using the FASTQ Clipper of FASTX-Toolkit (Pearson *et al.*, 1997). The remaining reads were filtered on the basis of quality score using Trimmomatic (v.0.32) (Bolger *et al.*, 2014) in the following steps: removal of adaptors, removal of low quality base calls and low quality regions from the starts and ends of reads, and removal of the read when the average Phred quality score per base was <10 by scanning of the reads with a 4 bp sliding window.

The STACKS pipeline (Catchen *et al.*, 2013) was used to detect SNPs from the sequencing data. USTACKS, CSTACKS, SSTACKS, and GENOTYPE programs were used to create libraries of loci. Detailed parameters are as follows: USTACKS, -t gzfastq -i -m 3 -M 3 -p 15 -d -r -f -o; CSTACKS, -b 1:M 3 -p 15 -d -r; SSTACKSm -b 1 -c -p 15; and GENOTYPE, -b 1 –P -r 1 -c -s -t CP; all other parameters used default values.

For the linkage analysis, a filtration step for SNPs with stringent conditions was executed according to the following criteria: (i) fragments with a missing rate <30%; and (ii) the segregation ratio of the SNP was 1:2:1.

### QTL mapping

MapQTL 5.0 (Ooijen, 2004) was used for QTL mapping with multiple QTL mapping (MQM). The detection of significantly associated markers as cofactors was conducted by automatic cofactor selection (backward elimination, *P*<0.05). Logarithm of odds (LOD) significance threshold levels were determined on the basis of 1000 permutations at significance levels of *P*<0.05. The location of each QTL was determined according to its LOD peak location and surrounding region. The percentage of the phenotypic variance explained by a QTL was estimated at the highest probability peak.

### BSR-seq analysis

BSR-seq, a method that combines bulked segregant analysis (BSA) and RNA-seq (Sanzhen *et al.*, 2012), was used to identify genetic markers linked to target genes. We divided the flower development stages of *D. chinensis* into six stages (stage S1–S6). Stage S1 corresponds to flower initiation; stages S2–S5 correspond to the sepal, petal, stamen, and carpel primordium formation stage, respectively; and stage S6 corresponds to the late flower development stage (Li *et al*., 2012). Flower buds from stages S1 to S6 of flower development from the two parent plants, from 30 single flower and from 30 double flower F_2_ individuals, were collected under a dissecting microscope and stored at –80 °C (six flower buds were collected from each plant, combined, and then used to prepare RNA). TRIzol reagent (Invitrogen) was used to extract total RNA. A library was constructed according to Illumina instructions and sequenced on a HiSeq 4000 sequencer. Raw RNA-seq reads were assessed for quality control, and low-quality sequences were removed using Trimmomatic v0.32 software with default parameters (Bolger *et al.*, 2014). STAR v2.4.0j was used to align high-quality reads to the reference sequence published in the Carnation DB (http://carnation.kazusa.or.jp) with default parameters. The Haplotype-Caller module in the software GATK v3.2-2 was used to perform variant calling with the default parameter (McKenna *et al.*, 2010). In addition, variants with the criteria of allele frequency difference (AFD) >0.8 and Fisher’s exact test *P*-value <1e−10 were considered to be putatively linked to the target gene.

### Selection of candidate genes for the double flower trait

To identify genes related to the double flower phenotype, we performed integrative analysis of QTL mapping and BSR-seq. First, the SNP sequences in the genetic map were mapped to a scaffold on the reference genome. The Burrows–Wheeler alignment (BWA) tool was used for alignment using default parameters; unique alignment was conducted between the SNP and the scaffold on the reference genome. A genetic map comprised of scaffolds was obtained and considered as the chromosome. Secondly, the identified SNPs associated with the double flower trait were mapped to the scaffold by BWA with default parameters, which were obtained from BSR-seq analysis. Finally, we analyzed the location of scaffolds obtained from BSR-seq in the genetic map comprised of scaffolds. According to the location of these candidate scaffolds in the genetic map, the region closely related to the double flower trait was identified, the genes in which were considered as candidate genes and were subjected to the following comprehensive analyses.

### Molecular, phylogenetic, and expression analyses

The full-length sequence and the coding sequence (CDS) of *DcAP2L* were cloned from the two parents, 10 single flower and 10 double flower F_2_ plants, and the full-length sequence of *DcAP2L* was cloned from different flower type varieties of *D. chinensis* (‘LH’, ‘CB’, and ‘HB’ are single flower phenotypes, and ‘DPD’, ‘DB’, and ‘ZX’ are double flower phenotypes). The putative protein sequence of *DcAP2L* from *D. chinensis* was compared with the protein sequence of euAP2 members from *Arabidopsis thaliana* (At), *Medicago truncatula* (Medrt), *Vitis vinifera* (Vv), *Solanum lycopersicum* (Sl), *Petunia hybrida* (Ph), and *Rosa chinensis* (Rc) (Supplementary Dataset S1). MUSCLE was used to align sequence with default parameters, and MEGA6 (Tamura *et al.*, 2013) was used to perform phylogenetic analysis with default parameters. The Neighbor–Joining (NJ) method was used to estimate evolutionary relationships with 2000 bootstrap replicates.

Two *DcAG* genes in the BSR-seq data were obtained, named *DcAGa* and *DcAGb*, and used for expression analysis. Quantitative real-time PCR was used to analyze the expression of *DcAP2L* and *DcAG* genes during flower development using RNA extracted from flower buds from stages S1 to S6. Quantitative real-time PCR analyses were performed as described previously (Zhang *et al*., 2018). *GAPDH* was used as the housekeeping gene. The primers used in these analyses are listed in Supplementary Table S2.

### Validating the miR172a–*DcAP2L* interaction *in vivo*

The core sequences of the miR172 family are highly conserved in plants. We used the miR172a precursor in Arabidopsis as a reference sequence to clone the fragment containing the sequence of the miR172a precursor in *D. chinensis*. The primers are listed in Supplementary Table S2. In order to validate the cleavage of *DcAP2L* by miR172, the fragment containing the sequence of pre-miR172a was cloned into an overexpression vector pICH86988 by Golden Gate cloning (Engler *et al.*, 2009, 2014), and the miR172 target site sequence of *DcAP2L* fused with the green fluorescent protein (GFP) reporter gene was then cloned into pICH86988. The vector was introduced into *Agrobacterium tumefaciens* (GV3101) followed by agroinfiltration into *Nicotiana benthamiana* leaves. The transient expression experiment was as described in Sparkes *et al.* (2006), and miR477a was cloned into the overexpression vector pICH86988 as a negative control.

## Results

### Observation of flower buds by scanning electron microscopy

**Fig. 1.**
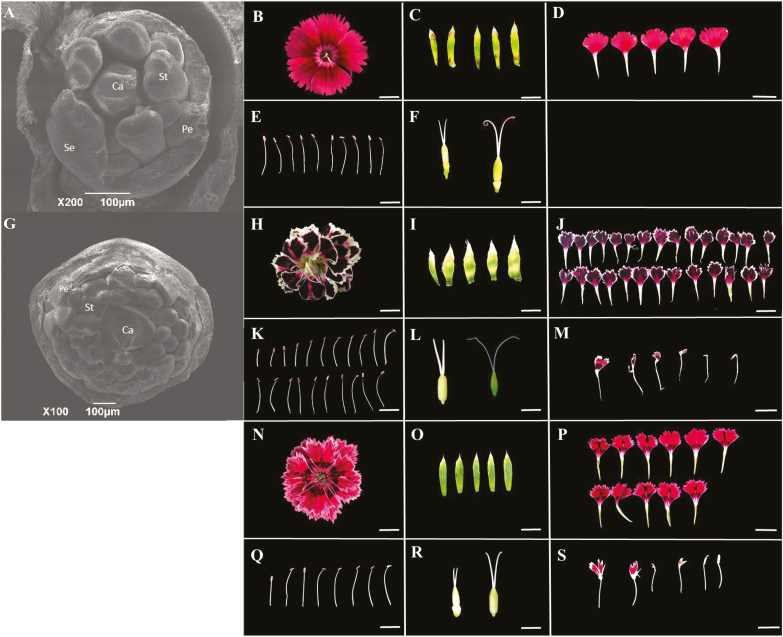
SEM imaging of flower buds from ‘MH’ (A) and ‘X4’ (G). Se, Pe, St, and Ca represent sepal, petal, stamen, and carpel, respectively. Scale bar=100 µm. View of the flower, sepals, petals, stamens, carpels, and the stamens of ‘MH’ (B–F), ‘X4’ (H–L), and of the F_1_ line (N–R) are shown. Note the presence of a petal–stamen chimera in ‘X4’ (M) and in the F_1_ hybrid (S). Young carpels (left) and mature carpels (right) are shown in (F), (L), and (R). (This figure is available in color at *JXB* online.)

SEM was used to compare the floral organs in single and double flower parents (Fig. 1A). The numbers of calyxes are the same in the two parents, while the numbers of petal and stamen primoridia in the double flower parent are considerably more than in the single flower parent (Fig. 1; Supplementary Table S3). The floral primordium surface area of ‘MH’ was ~0.081±0.003 mm^2^, while that of ‘X4’ was ~0.442±0.038 mm^2^. The ‘MH’ flower is composed of five petals, 10 stamens, and no chimera petal–stamen could be observed, while the ‘X4’ flower is composed of 25.43±3.18 petals, 13.62±2.26 stamens, and 3.43±1.93 chimera petal–stamen organs (Fig. 1; Supplementary Table S3). The single flower parent is composed of an average of 22 floral organs, while the ‘X4’ flower has many more floral organs, ~55 (Supplementary Table S3). This increase in total organ number in the double flower parent is consistent with an increase in its flower primordium size.

### Development of an F_2_ mapping population

To identify the nature of the mutation responsible for double flower formation, we generated an F_2_ mapping population in *D. chinensis* by an intraspecific cross between the single-flowered line ‘MH’ and the double-flowered line ‘X4’. The F_1_ hybrids have semi-double flowers containing 9.52±1.65 petals, 8.54±2.33 stamens, and 2.34±1.58 stamens converted to petals (Fig. 1; Supplementary Table S3). The F_1_ generation was self-crossed and 400 F_2_ individuals were generated. The data of agronomic traits for the parents and 400 F_2_ individuals were measured in 2016. Of the 400 F_2_ individuals, 140 with similar growth potential as well as the two parents were selected for genotyping and mapping.

### ddRAD-seq library sequencing, SNP genotyping, and genetic map construction

A total of 142 ddRAD-seq libraries (two parents and 140 individuals of the F_2_ progeny) were constructed. After data filtering, ~56.12 Gb of data containing 381 774 866 paired-end reads were generated, with 150 bp average length for each read. The parent ‘MH’ and ‘X4’ libraries contained 5 287 064 and 5 222 674 filtered reads, respectively (Supplementary Table S4). The 140 F_2_ progeny libraries contained a total of 371.26 million filtered reads, with an average of 2.65 million reads per offspring, which corresponded to 520.52 Mb. The mean sequencing depths of the parent and the progeny genomes were 24.5- and 9.6-fold, respectively (Supplementary Table S4). The high depth of coverage at the locus-specific sequences provided confidence in the accuracy of the SNP marker discovery.

After filtering of the raw reads, a total of 59 841 SNP markers were acquired from cleans reads. Following the strict filtration steps, 2353 high-quality SNPs were finally obtained. More than half of the identified SNPs were transition-type SNPs, and the transversion-type SNPs containing C/G, G/T, C/A, and A/T substitutions were detected at rates of 6.97, 8.59, 9.77, and 13.77%, respectively (Supplementary Table S5).

**Fig. 2.**
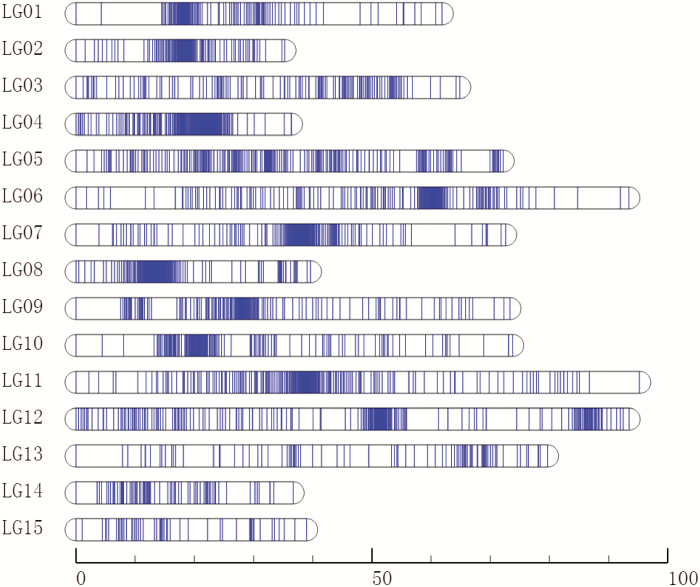
High-density genetic map of *D. chinensis*. The horizontal bar represents a linkage group and each line indicates an SNP marker. The bottom number represents units of centiMorgans (cM).

The 2353 SNPs, distributed across 15 LGs, were used to construct the high-density genetic map (Fig. 2). The total length of the genetic map was 967.54 cM, with an average of 0.41 cM between two neighboring SNPs and an average of 156 SNPs per LG. The length of each LG ranged from 35.29 cM (LG2) to 95.24 cM (LG11), with an average size of 64.50 cM. LG11 was the largest, containing 217 SNPs with an average intermarker distance of 0.44 cM. By comparison, the smallest LG, LG2, contained 118 SNPs with an average intermarker distance of 0.30 cM (Table 1). The ‘Gap ≤5 cM’ value ranged from 71.86% to 100% (average of 92.93%) across the 15 LGs, indicating that LG01 has the largest interval compared with other LGs (Table 1).

**Table 1.** Description on basic statistics of 15 linkage maps

LG ID	No. of SNPs	Total distance (cM)	Density (cM per locus)	Max gap (cM)	Gap ≤5 cM
LG01	132	61.88	0.47	10.23	71.86%
LG02	118	35.29	0.30	3.38	100%
LG03	130	64.86	0.50	3.11	100%
LG04	204	36.39	0.18	3.25	100%
LG05	210	72.19	0.34	4.83	100%
LG06	208	93.45	0.45	7.23	85.93%
LG07	205	72.61	0.35	7.36	89.86%
LG08	178	39.63	0.22	3.43	100%
LG09	170	73.36	0.43	7.54	89.72%
LG10	154	73.81	0.55	5.12	93.06%
LG11	217	95.24	0.44	8.46	91.11%
LG12	213	93.49	0.44	5.39	88.72%
LG13	87	79.71	0.91	7.86	83.72%
LG14	75	36.67	0.49	4.02	100%
LG15	52	38.96	0.75	3.38	100%
Total	2353	967.54	–	–	–
Average	156	64.50	0.41	–	92.93%

‘Gap ≤5 cM’ indicates the percentage of gaps in which the distance between adjacent markers is smaller than 5 cM.

### QTL mapping for horticultural traits

QTL analysis was performed for 12 important horticultural traits in *D. chinensis*. Nine of these traits, namely double flower trait (DFT), stamen number (SN), plant height (PH), plant width (PW), major stem diameter (SD), branch number (BN), stem color (SC), calyx color (CC), and style color of the pistil organ (PC), were significantly associated with QTL regions on the genetic map (Supplementary Fig. S3). No significant QTL region was found for leaf length, leaf width, or leaf with wax. Significance tests of these agronomic characters between parents and character statistics of parents and offspring are shown in Supplementary Table S6; DPS7.05 software was used for character statistics.

**Fig. 3.**
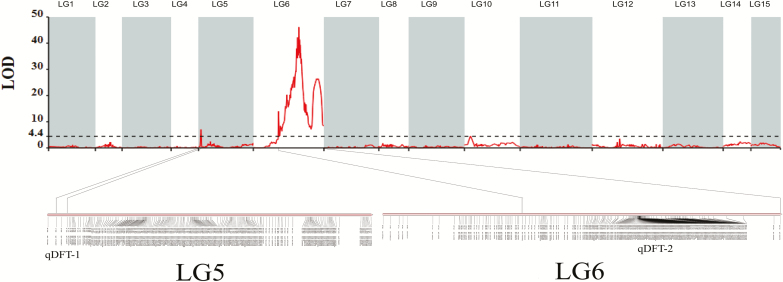
Genetic location of QTLs for the double flower trait. The horizontal dashed line represents a linkage group-wise logarithm of odds (LOD) significance threshold of 4.4. qDFT-1 and qDFT-2 are QTL regions for the double flower trait with a higher LOD value than the LOD significance threshold. (This figure is available in color at *JXB* online.)

Two major QTL regions, qDFT-1 and qDFT-2, were identified for the double flower trait, located in LG5 and LG6, respectively (Fig. 3). qDFT-1 was located at 1.88–4.08 cM in LG5, accounting for 20.7% of the phenotypic variation (Table 2). qDFT-2 was detected in LG6 at 32.87–93.45 cM, accounting for 78.1% of phenotypic variation (Table 2). The peak LOD value of qDFT-2 was far higher than that of qDFT-1, thereby indicating that the locus in LG6 was strongly associated with the double flower type. A total of 31 QTL regions were detected for the other eight horticultural traits (Table 2). Three QTLs (qSN-1, qSN-2, and qSN-3) for stamen number were detected across LG9 and LG15. Eight QTLs for stem diameter were identified, all located in LG11. For the plant height trait, two QTLs (qPH-1 and qPH-2) were located on LG7 and a further two QTLs (qPH-3 and qPH-4) were detected on LG11. For the color-related traits, the highest LOD scores for the three associated QTLs were mapped to the same position which corresponded to a 64.86cM region at the end of LG3 (Table 2; Supplementary Fig. S4).

**Table 2.** Statistics of QTLs for nine agronomic traits in the F_2_ mapping population

QTL	Linkage map	Interval (cM)	Peak position (cM)	Peak LOD	Exp%	No. of markers
qDFT-1	5	1.88–4.08	3.08	7.04	20.7	2
qDFT-2	6	32.87–93.45	60.26	46.12	78.1	169
qSN-1	9	50.68–52.21	51.06	4.30	13.2	5
qSN-2	9	64.71–65.83	65.12	4.30	13.2	3
qSN-3	15	37.98–38.96	38.96	4.52	14.6	1
qPH-1	7	0–53.59	27.83	11.32	31.1	194
qPH-2	7	54.91–55.69	55.35	6.52	19.4	3
qPH-3	11	33.61–34.30	33.99	4.84	14.7	3
qPH-4	11	36.38–36.67	36.53	4.49	13.8	3
qPW-1	11	33.27–33.99	33.61	4.30	14.2	3
qSD-1	11	34.9–35.53	35.23	5.32	16.1	3
qSD-2	11	37.07–37.25	37.15	4.36	13.5	3
qSD-3	11	37.42–37.68	37.48	4.83	14.7	4
qSD-4	11	37.77–37.97	37.88	4.42	13.5	3
qSD-5	11	38.03–38.13	38.08	4.25	13.1	3
qSD-6	11	39.25–39.31	39.26	4.24	13	3
qSD-7	11	39.43–39.65	39.54	4.57	14	4
qSD-8	11	39.78–39.96	39.87	4.22	13	3
qSC-1	3	41.24–42.13	41.59	4.82	15.1	5
qSC-2	3	42.43–64.86	64.86	17.56	44	54
qSC-3	6	0–4.69	3.77	4.42	14.4	4
qCC-1	3	28.27–29.89	28.89	4.31	14	2
qCC-2	3	31.72–34.29	32.72	4.62	15.5	2
qCC-3	3	35.31–36.15	35.82	4.33	13.3	3
qCC-4	3	37.13–64.86	64.86	15.33	39.7	69
qPC-1	1	2.00–10.25	6.25	6.73	34.6	1
qPC-2	3	27.38–29.89	28.27	5.35	16.1	5
qPC-3	3	31.72–64.86	64.86	27.40	62.1	76
qBN-1	3	10.93–15.79	14.21	7.52	25.5	10
qBN-2	3	23.46–23.93	23.80	5.50	16.5	3
qBN-3	3	28.89–30.33	29.89	5.41	22.8	2

DFT, SN, PH, PW, SD, BN, SC, CC, and PC represent double flower trait (petal number), stamen number, plant height, plant width, major stem diameter, branch number, stem color, calyx color, and style color of the pistil organ, respectively.

### Identification of candidate genes for the double flower trait

**Fig. 4.**
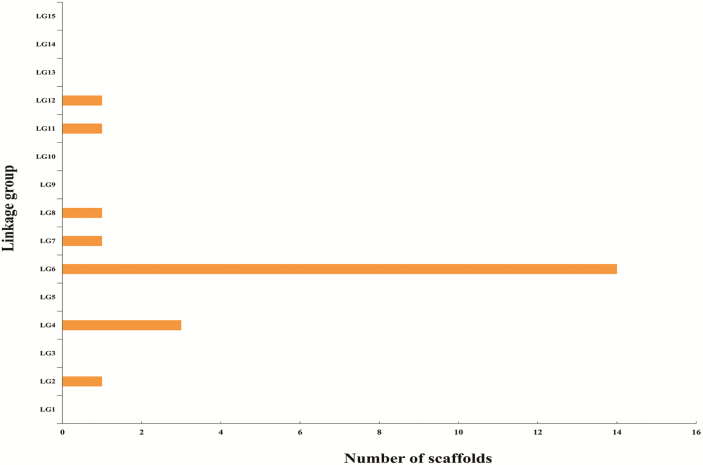
Distribution of candidate scaffolds associated with the double flower trait on linkage groups of *D. chinensis*. (This figure is available in color at *JXB* online.)

We obtained a genetic map with scaffolds by mapping the SNPs in the genetic map to the reference genome. According to BSR-seq analysis, 479 683 high-quality variants (SNPs and InDels) were identified. A total of 126 SNPs distributed in 42 scaffolds were identified to be associated with the double flower trait. Due to the fact that the reference genome lacked chromosome information, the observed SNPs were distributed across scaffolds rather than on chromosomes. The distribution of the 42 scaffolds in LGs was analyzed. Twenty scaffolds were detected, among which 14 were distributed in LG6, thus supporting the validity of QTL mapping and BSR-seq analysis (Fig. 4). By analyzing the position of these 14 scaffolds in LG6, seven of them were located near (~2 cM) the peak position of the qDFT-2 region which was considered as the candidate region. A total of 54 scaffolds containing 327 genes (Supplementary Dataset S2) were obtained from this region using the reference genome. Among these genes, one encodes an *AP2* domain-containing transcription factor. This gene was named *DcAP2L*. *AP2* is an A-class gene, known as a floral identity gene, not only represses the C-class gene *AG*, but also regulates the expression of the B-class genes *APETALA3* and *PISTILLATA*, and the E-class gene *SEPALLATA3* (Krogan *et al.*, 2012).

### Sequence analysis for *DcAP2L*, and phylogenetic and expression analysis

**Fig. 5.**
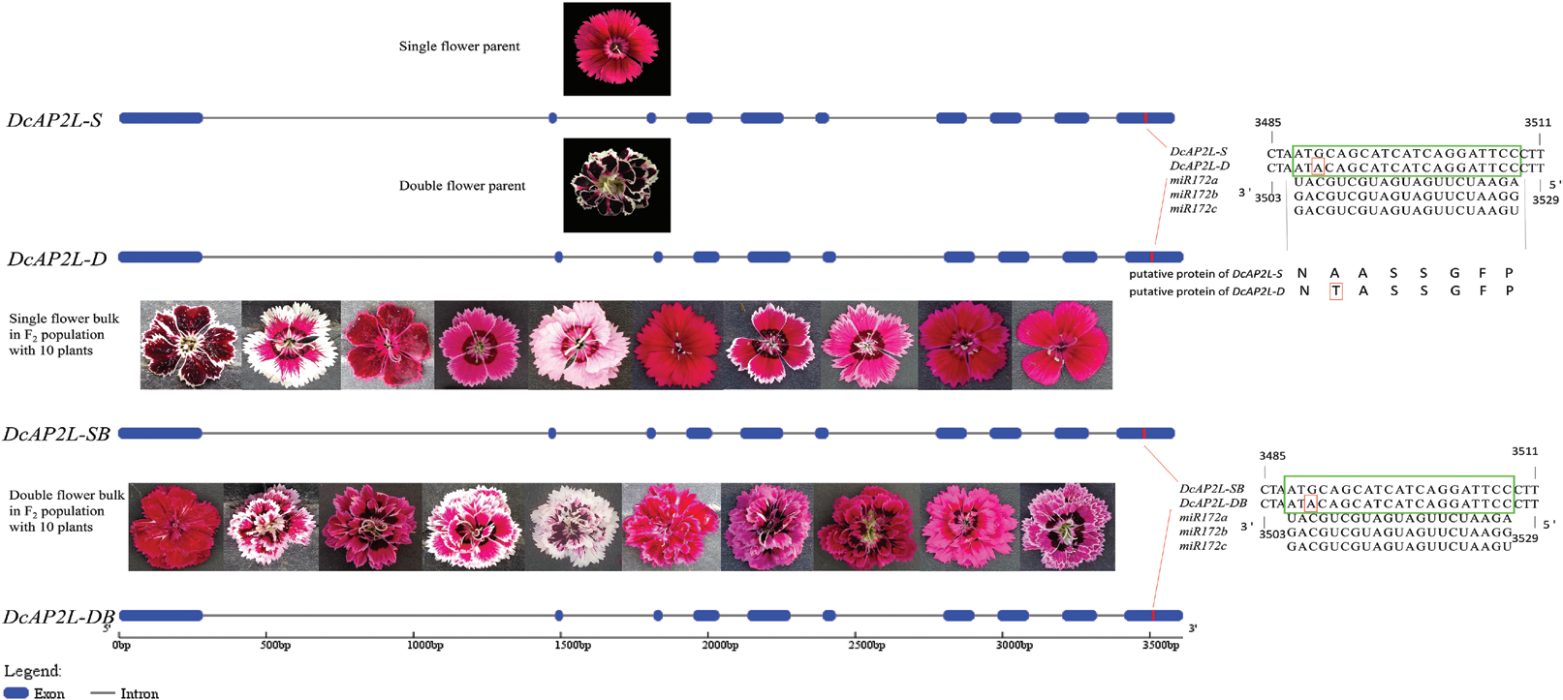
Gene structure and miRNA-binding site alignments of *DcAP2L* between two parents and two bulks (containing 10 single flower and 10 double flower plants, respectively) in the F_2_ population. *DcAP2L-S*, *DcAP2L-D*, *DcAP2L-SB*, and *DcAP2L-DB* represent the sequence of *DcAP2L* in ‘MH’, ‘X4’, single flower bulk, and double flower bulk of F_2_, respectively. The putative miR172-binding site is marked in the last exon of *DcAP2L*, and the large box on the right aligns with the sequences of miR172a, b, and c from peach (Zhu *et al.*, 2012). A single nucleotide polymorphism in the miRNA-binding site is found in *DcAP2L*, marked with a small box within the large box, which leads to a missense mutation (small box) in the sequence of the putative amino acid. The sequence of *DcAP2L* in the double flower bulk of F_2_ is the same in the miR172 target site compared with that in single flower bulk, indicating that this SNP exists in both single flower and double flower plants in *D. chinensis.* (This figure is available in color at *JXB* online.)

**Fig. 6.**
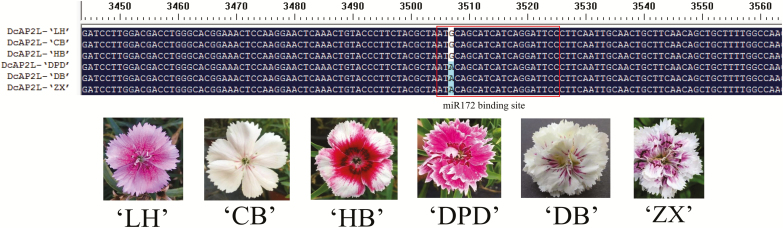
Partial sequence alignment of *DcAP2L* in different flower type varieties of *D. chinensis*. *DcAP2L*-‘LH’, D*cAP2L*-‘CB’, *DcAP2L*-‘HB’, *DcAP2L*-‘DPD’, *DcAP2L*-‘DB’, and *DcAP2L*-‘ZX’, represent the full-length sequence of *DcAP2L* in ‘LH’, ‘CB’, ‘HB’, ‘DPD’, ‘DB’, and ‘ZX’, respectively. Sequence differences are shown at the location of base pair 3507. The miR172 target site is marked with a box. ‘LH’, ‘CB’, ‘HB’, ‘DPD’, ‘DB’, and ‘ZX’ are different varieties collected by our group. (This figure is available in color at *JXB* online.)

The full-length sequence and the CDS of *DcAP2L* were cloned from single and double flower parent plants. Sequence alignments of the full-length sequence of *DcAP2L* from the two parents identified two InDels and 13 SNPs, among which six SNPs were found in the CDS region (Supplementary Figs S1, S2). Among these point mutations located in the coding region, one of them is a missense mutation that lies within the miR172 target site in exon 10 (Fig. 5; Supplementary Figs S1, S2, S5). Such a mutation is thus expected to exist in all double flower-type *D. chinensis*. To test this hypothesis, we obtained the full-length sequence of *DcAP2L* from other varieties with different flower type, and the full-length sequence and CDS of *DcAP2L* from F_2_ single and double flower bulks. Interestingly, this point mutation was also within the miR172 cleavage site of *DcAP2L* (Fig. 6; Supplementary Figs S2, S3).

**Fig. 7.**
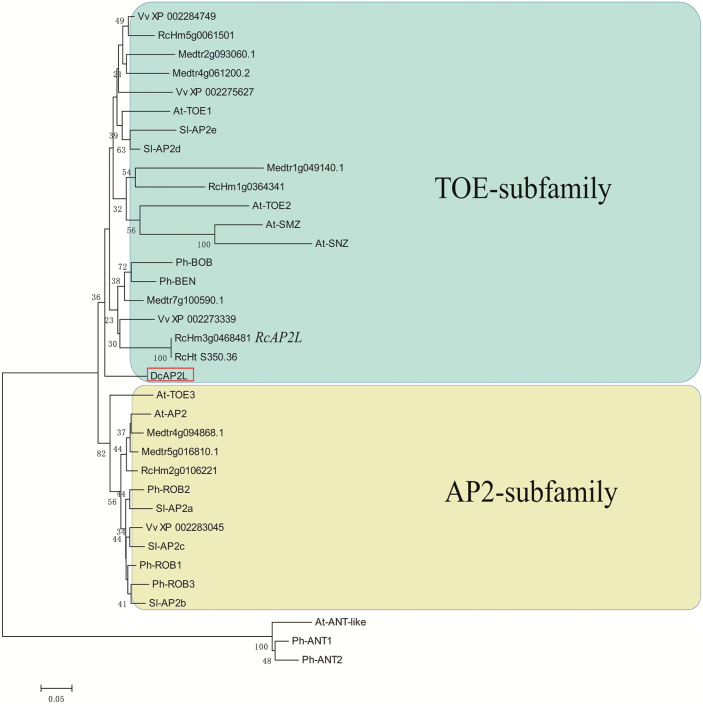
Neighbor–Joining (NJ) tree based on the alignment of *DcAP2L* and euAP2 members of *Arabidopsis thaliana*, *Medicago truncatula*, *Vitis vinifera*, *Solanum lycopersicum*, *Petunia hybrida*, and *Rosa chinensis*. *DcAP2L* belongs to the euAP2 family and the TOE subfamily. The NJ method is used to estimate evolutionary relationships with 2000 bootstrap replicates. (This figure is available in color at *JXB* online.)

**Fig. 8.**
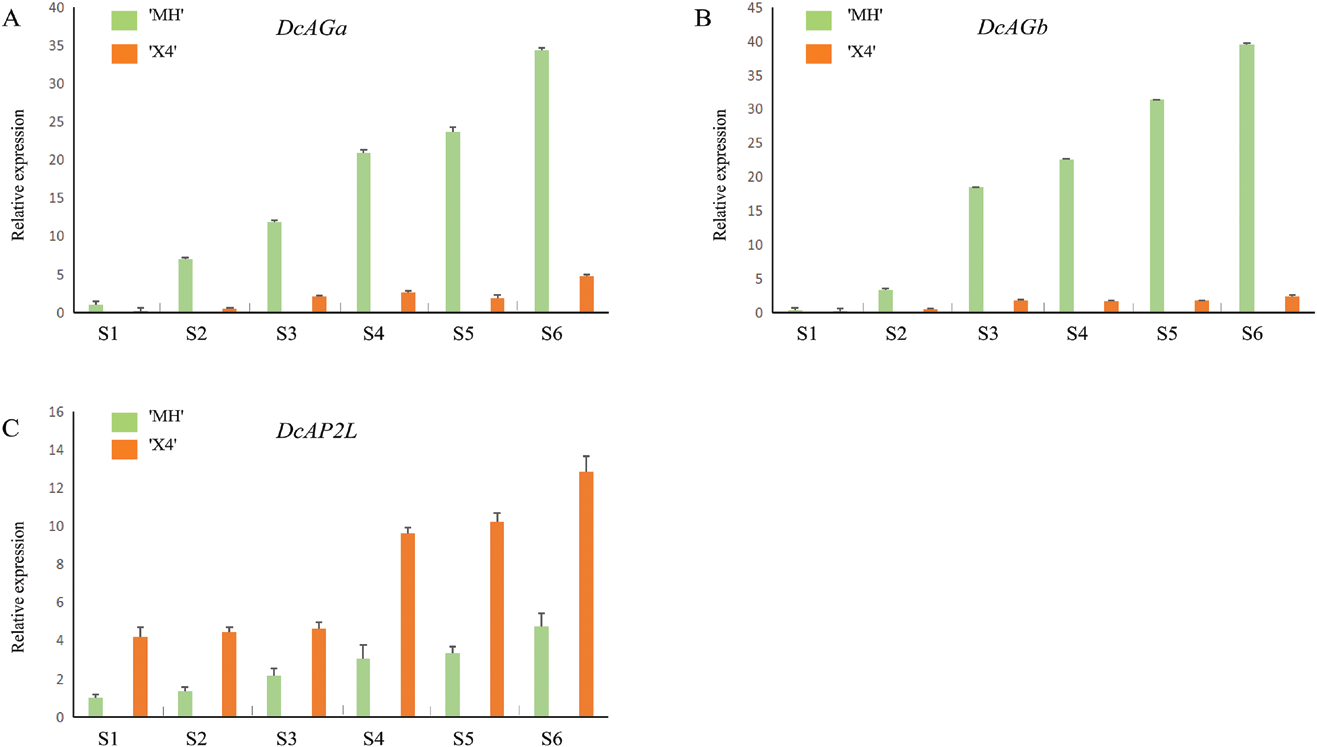
The expression analysis of *DcAG* genes (A and B) and *DcAP2L* (C) in the process of flower development. Stage S1 represents the stage of floral initiation, stages S2–S5 correspond to sepal, petal, stamen, and carpel primordium formation, and stage S6 represents the late flower development stage. (This figure is available in color at *JXB* online.)

Phylogenetic analysis showed that *DcAP2L* is related to the TOE subfamily of the euAP2 family (Fig. 7). *DcAP2L* is closely related to the rose *RcAP2L* that we previously showed to be associated with double flower formation in roses, probably through the regulation of expression of the rose *AGAMOUS* (*AG*) (Dubois *et al.*, 2010; Francois *et al.*, 2018). A-class genes have an antagonistic role toward the expression of the C-class gene *AG* (Bowman *et al.*, 1991; Drews *et al.*, 1991). The mutation in the miR172 target site is expected to reduce the predicted energy of the interaction of miR172 with its target sequence, which may have an effect on transcript levels of *DcAP2L*, thus we compared the expression of *DcAP2L*, *DcAGa*, and *DcAGb* between the double flower and single flower parents. Both *DcAGa* and *DcAGb* exhibited lower expression in the double flower parent compared with the single flower parent at flower development stages S1–S6 (Fig. 8A, B). Moreover, the observed low expression of *DcAGa* and *DcAGb* correlated with high expression levels of *DcAP2L* in the double flower parent (Fig. 8C), suggesting that expression of *DcAG* genes could be inhibited by *DcAP2L* in the double flower parent.

### Validation of miR172a–*DcAP2L* interaction *in vivo*

**Fig. 9.**
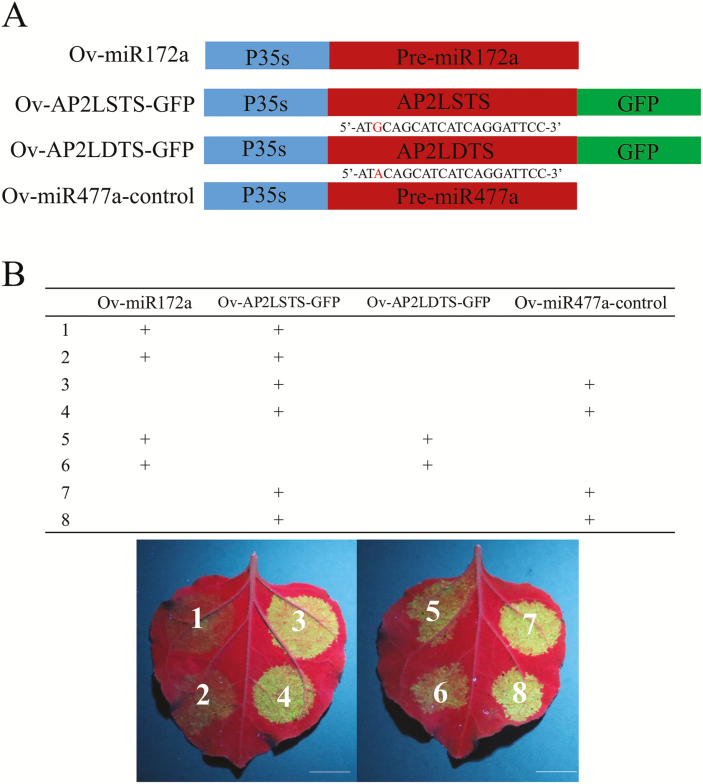
Validation of the miR172a–*DcAP2L* interaction *in vivo*. (A) Four vectors are constructed for the transient expression experiment. Ov-miR172a, Ov-AP2LSTS–GFP, Ov-AP2LDTS–GFP, and Ov-miR477a-control represent overexpressing pre-miR172a, overexpressing the GFP gene carrying the miR172a target site of *DcAP2L* in the single flower parent, overexpressing the GFP gene carrying the miR172a target site of *DcAP2L* in the double flower parent, and overexpressing pre-miR477a, respectively. (B) The decreased expression of GFP shows that miR172a could target *DcAP2L* in *D. chinensis*. Leaves of tobacco are photographed on the third day after injection under UV light (wavelength=365 nm). The experiment was repeated three times. The miR477a was cloned into the overexpression vector pICH86988 as a negative control. Scale bar=1 cm. (This figure is available in color at *JXB* online.)

The sequence of the fragment containing pre-miR172a is given in Supplementary Table S7. The interaction of miR172a and *DcAP2L* was validated by transient expression in tobacco leaf. Two target sites of miR172 in *DcAP2L* of two parents were fused with the GFP gene and then inserted into the overexpression vector pICH86988 (Fig. 9A). Compared with the negative group, the decreased expression of GFP in the Ov-AP2LSTS–GFP and Ov-AP2LDTS–GFP group showed that miR172a could target *DcAP2L* in *D. chinensis* (Fig. 9B), which was consistent with the findings of a previous study (Chen, 2004). Moreover, the decrease of GFP expression in the Ov-AP2LDTS–GFP group was very weak when compared with the Ov-AP2LSTS–GFP group (Fig. 9B), which might indicate that the efficiency of miR172a for cleaving *DcAP2L* in the double flower parent was lower than that in the single flower parent.

## Discussion

### Description of the double flower trait in *D. chinensis*

The first report of the double flower phenotype in the *Dianthus* genus dates back to 1917, where the double-flowered plants were shown to contain a variable number of extra petaloid structures because of a petalody of the androecium (Saunders, 1917). Caryophyllales are valued as pentamerous ornamental plants (Decraene *et al.*, 1998) and are typically considered as a representative plant for the study of the transition between pentamerous and trimerous flower forms (Soltis *et al.*, 2003). The wild-type *D. chinensis* represents a typical pentamerous plant with five calyxes, five petals, and 10 stamens. The single flower parent ‘MH’ used in this study belongs to the standard pentamerous flower type, whereas the double flower parent ‘X4’ displays a developmental pattern which disorganizes the formation of the pentamerous flower organ. The study of the double flower type in *D. chinensis* represents an excellent foundation for a general theory to explain the pathways which result in loss of the pentamerous flower organ developmental pattern. It is reported that in most double flower species, the increase in petal number is a result of a homeotic conversion of stamens into petals, thus with a decrease in stamen number (Bowman *et al.*, 1989; Yellina *et al.*, 2010; Bendahmane *et al.*, 2013). In *A. thaliana*, mutation of the C-function *AG* gene results in homeotic conversion of stamens to petals, which leads to a decrease of stamen number and an increase of petal number. In many species such as *Rosa* sp., *Thalictrum thalictroides*, and *Cyclamen persicum*, the decrease in expression of *AG* in the third whorl leads to a homeotic conversion of stamens into petals and formation of the double flower (Dubois *et al.*, 2010; Galimba *et al.*, 2012; Tanaka *et al.*, 2013). In *D. chinensis*, the number of both stamens and petals was increased in the double flower parent (Fig. 1; Supplementary Table S1), suggesting that in contrast to many other species, the double flower formation in *Dianthus* is probably not associated with homeotic conversion of stamens to petals, but it is likely to be related to the primordial numbers of stamens and petals. This is further supported by the fact that the surface of flower primordia is ~2.5 times larger in the double flower, compared with the simple flower *Dianthus* (Fig. 1; Supplementary Table S3)

### Multiple agronomic trait QTL mapping in *Dianthus* spp.

Previously, a high-density genetic map for *D. caryophyllus*, consisting of 2119 RAD and 285 SSR markers, was constructed by using ddRAD-seq, but no QTLs or candidate genes were detected in the study (Yagi *et al.*, 2016). Here, we develop a high-quality and high-density genetic map suitable for QTL mapping and identification of candidate genes in the *Dianthus* genus. A total of 12 desirable ornamental traits were targeted for QTL analysis using our high-density genetic map and the segregation data of the parent line and F_2_ population. Nine of the addressed traits corresponded to significant QTL regions on the genetic map. A total of 31 QTL regions were detected on LG1, LG3, LG5, LG6, LG7, LG9, LG11, and LG15, and accounted for nine ornamental traits. In the genus *Dianthus*, the first QTL analysis was reported in *D. caryophyllus* on the resistance to bacterial wilt caused by *Burkholderia caryophylli* (Yagi *et al.*, 2006). Thereafter, an SSR-based genetic linkage map was constructed for QTL analysis for resistance to bacterial wilt in *D. caryophyllus* (Yagi *et al.*, 2012). Since then, no or very little, information on QTLs associated with other agronomic traits have been reported in *Dianthus*. In the past few years, only a limited number of examples have been reported on successful QTL mapping in other ornamental plants. A genetic linkage map comprising 75 SSR and six AFLP markers spanning 359.1 cM across seven LGs was developed for petunia and was used to identify 24 QTLs for 10 crop timing and quality traits (Vallejo *et al.*, 2015). A high-density SNP integrated genetic map with good genome coverage of *Dendrobium* is constructed, which contains 8573 SLAF (specific locus amplified fragment) markers covering 19 LGs. QTL mapping for stem total polysaccharide contents (STPCs) was conducted, and five QTLs related to STPCs were identified (Lu *et al.*, 2018). In the present study, we develop a high-density genetic map with mapping of QTLs for many agronomic traits that have important breeding significance within the *Dianthus* genus.

### *DcAP2L* may influence the formation of double flowers in *D. chinensis*

High-throughput SNP genotyping data, BSR-seq analysis, and the available reference genome were used for QTL fine mapping and detection of candidate genes. Through integrated analysis of these various datasets, we identify an A-class gene *DcAP2L* which lies within the DFT QTL as candidate gene for the double flower formation in *D. chinensis*. In *D. caryophyllus*, two SSR markers (CES0212 and CES1982) were tightly linked to the *D*_*85*_ locus which controls flower type (Yagi *et al.*, 2014). Interestingly, CES0212 is mapped to the scaffold which harbors *DcAP2L*. Phylogenetic analysis shows that *DcAP2L* belongs to the euAP2 family and is more closely related to the TOE subfamily (Fig. 7). Sequence comparison shows that *DcAP2L* in double-flowered *Dianthus* diverges for that in the simple flower by an SNP in the miR172 target site. The miR172 target site, an important characteristic of euAP2 family members, is essential for their post-transcriptional regulation by miR172 (Shigyo *et al.*, 2006). In Arabidopsis, an *AP2* lacking the miR172 target site is resistant to post-transcriptional targeted degradation by miR172, leading to its maintenance in the center of the meristem, and in turn to continuous inhibition of *AG* expression (Zhao *et al.*, 2007; Wollmann *et al.*, 2010). Similarly, overexpressing an miR172-resistant *TOE3* gene in Arabidopsis results in indeterminate flowers and decreased expression of *AG* in flowers, indicating that other members of the euAP2 family also have an important role in flower development and an antagonistic role in regulating the expression of *AG* (Jung *et al.*, 2014).

Moreover, in Arabidopsis, loss of function of *AP2* leads to ectopic expression of *AG* in the first and second whorls, which also results in homeotic conversion of sepals into carpel-like structures and loss of petals (Bowman *et al.*, 1991; Drews *et al.*, 1991). *AP2* antagonizes the transcriptional activity of *AG* that also results in promoting the maintenance of the floral stem cell fate (Huang *et al.*, 2017). In agreement with these published data, the expression of *DcAGa* and *DcAGb* inversely correlated with that of *DcAP2L* (Fig. 8) in *D. chinensis*. These data are in agreement with the hypothesis that overaccumulation of mutated *DcAP2L*, putatively resistant to miR172-mediated degradation, leads to continuous repression of *DcAG* genes and that this could be at the origin of the double flower. Our findings are in agreement with previously reported data in the rose, which show that in double flower roses an allele of the *RcAP2L* gene lacking the miR172 target site because of a transposable element insertion is responsible for the double flower formation phenotype (Francois *et al*., 2018). In peach, a similar mechanism involving a deletion of the target site for miR172 in a candidate gene encoding an euAP2 transcription factor is also identified to associate with double flower formation (Gattolin *et al.*, 2018). Our data corroborate these reports in the rose and peach, and provide another piece of evidence that mutation of *AP2*-like genes is at the origin of double flower formation in many species.

**Fig. 10.**
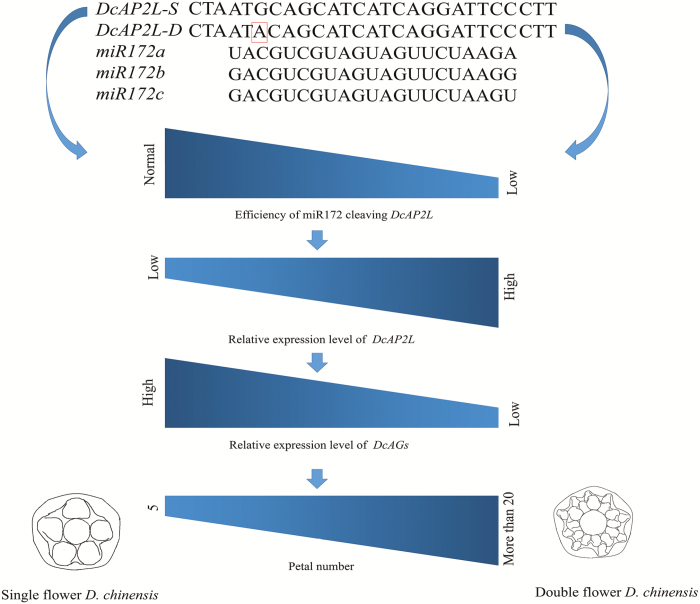
The model is established to explain how the mutation in the miR172 target site of *DcAP2L* could lead to double flower formation in *D. chinensis*. In the double-flowered *D. chinensis*, the mutation in the miR172 target site of *DcAP2L* results in lower efficiency of miR172 cleaving *DcAP2L*, which results in higher expression of *DcAP2L*, compared with single-flowered *D. chinensis*. It, in turn, inhibits the expression of *DcAG* genes in double-flowered *D. chinensis*, which leads to double flower formation. *DcAP2L-S* and *DcAP2L-D* represent the sequence of *DcAP2L* aligned with the sequences of miR172a, b, and c from peach (Zhu *et al.*, 2012), in ‘MH’ and ‘X4’, respectively. (This figure is available in color at *JXB* online.)

In *D. chinensis*, an SNP in the miR172 target site of *DcAP2L* is detected in the double flower parent. This opens up the question of whether such an SNP could be sufficient to affect targeting of *DcAP2L* by miR172. We validate that miR172a could target the *DcAP2L* in *D. chinensis* (Fig. 9B) by a transient expression experiment, and the results might indicate that the efficiency of miR172a for cleaving *DcAP2L* in the double flower parent is lower than that in the single flower parent. This situation in *D. chinensis* may be similar to that in other species. In Arabidopsis, it is reported that a six nucleotide mismatch mutation of the miR172 target site in *AP2* (15 of 21 nucleotides) leads to an enlarged floral meristem, loss of floral determinacy, and the transformation of stamens to petals (Chen, 2004). In *Picea abies*, *PaAP2L2* (14 of 21 nucleotides) exhibits lower conservation of the miR172 target sequence than *PaAP2L1* and *PaAP2L3* (20 of 21 nucleotides). Arabidopsis plants overexpressing *PaAP2L2* are stunted and flowered later than wild-type plants. Flowers of *PaAP2L2* transformants which display more and larger petals, more stamens, and a shorter pistil, show the most severe phenotype (Nilsson *et al.*, 2007). However, in wheat, the *Q* allele (20 of 21 nucleotides), which exhibits a single mismatch in the miR172 target site of *AP2*, is reported to have a weaker interaction affinity for miR172 than the *q* allele (21 of 21 nucleotides), which results in increased *Q* allele expression and the formation of ectopic florets and spikelets (Debernardi *et al.*, 2017; Greenwood *et al.*, 2017). These published data and our data support the following model. Compared with single flower *D. chinensis*, the mutation in the miR172 cleavage site of *DcAP2L* in double flower *D. chinensis* results in lower efficiency of miR172 cleaving *DcAP2L*, which leads to higher expression of *DcAP2L*. The expression of *DcAG* genes is inhibited by *DcAP2L* in double flower *D. chinensis*, which leads to double flower formation (Fig. 10).

According to the observation of SEM and expression analysis of *DcAP2L* and *DcAG* genes, we speculate that mutation of *DcAP2L* in double-flowered plants could influence flower organ identity and the size of the floral meristem. In Arabidopsis, *AP2* promotes *WUSCHEL* (*WUS*) expression and stem cell maintenance, while *AG* represses *WUS* expression to elicit stem cell termination (Yumul *et al.*, 2013). Compared with the single flower parent, the SNP in the miR172 target site of *DcAP2L* leads to the higher expression of *DcAP2L* which results in lower expression of *DcAG* genes in the double flower parent. This could have a positive effect on promoting *WUS* in the floral meristem, which leads to more floral organs and a larger meristem. Therefore, we believe that decreased expression of *DcAP2L* may affect the *WUS*–*AG* feedback regulation loop, which leads in turn to formation of extra petals via homeotic conversion of stamens into petals and also an increased number of floral organs.

## Supplementary data

Supplementary data are available at *JXB* online

Fig. S1. Sequence alignment of *DcAP2L*.

Fig. S2. Sequence alignment of the CDS of *DcAP2L*.

Fig. S3. Flower and stem color characteristics of the two parents.

Fig. S4. Genetic location of QTLs for color-related traits.

Fig. S5. Sequence alignment of the putative amino acid sequence of *DcAP2L*.

Table S1. Evaluation criteria for agronomic traits.

Table S2. Primers for quantitative PCR and cloning.

Table S3. Number of floral organs of ‘MH’, ‘X4’, and F_1_ plants in *D. chinensis*.

Table S4. Summary of sequence data from parents and 140 F_2_ progenies.

Table S5. Type of variation of the 2353 identified SNP markers.

Table S6. Character statistics of agronomic characters.

Table S7. Sequence of the fragment containing the sequence of pre-miR172a of *D. chinensis*.

Dataset S1 Sequence of euAP2 members used for phylogenetic analysis.

Dataset S2. Sequence of 2353 SNPs for the genetic linkage map.

Dataset S3. Genes in the qDFT-2 region tightly linked to the double flower trait.

erz558_suppl_Supplementary_Tables_Figures

erz558_suppl_Supplementary_Data_1

erz558_suppl_Supplementary_Data_2

erz558_suppl_Supplementary_Data_3

## Data Availability

The FASTQ files of the sequencing data were deposited to NCBI with accession numbers SRP152616 and SRP193022.
