# Supplementary material for: Mapping a double flower phenotype-associated gene DcAP2L in Dianthus chinensis
Source: J Exp Bot. 2020 Jan 28;71(6):1915–27. doi: 10.1093/jxb/erz558 (PMC7242084; doi:10.1093/jxb/erz558)
Supplement: erz558_suppl_Supplementary_Tables_Figures [file erz558_suppl_supplementary_tables_figures.pdf]

Mapping a double flower phenotype associated gene *DcAP2L* in *Dianthus chinensis* by using Reduced-Representation Genome Sequencing and BSR-seq analysis

Qijian Wang[1,2], Xiaoni Zhang[1,2], Shengnan Lin[1,2], Shaozong Yang[1,2], Xiuli Yan[1,2], Mohammed Bendahmane[1,3], Manzhu Bao[1,2], and Xiaopeng Fu[1,2,*]

[1]Key Laboratory of Horticultural Plant Biology, College of Horticulture and Forestry Sciences, Huazhong Agricultural University, Wuhan 430070, China; qjwang@webmail.hzau.edu.cn (Q.W.); zhangxiaoni@webmail.hzau.edu.cn (X.Z.); yangshaozong@webmail.hzau.edu.cn (S.Y.); mzbao@mail.hzau.edu.cn (M.B.); Yanxiuli@webmail.hzau.edu.cn (X.Y.); linshengnan@webmail.hzau.edu.cn (S.L.);

[2]Key Laboratory of Urban Agriculture in Central China (pilot run), Ministry of Agriculture, Wuhan 430070, China

[3]Laboratoire Reproduction et Development des Plantes, INRA-CNRS-Lyon1-ENS, Ecole Normale Supérieure de Lyon, Lyon 520074, France; mohammed.bendahmane@ens-lyon.fr

[*]Correspondence: fuxiaopeng@mail.hzau.edu.cn; Tel.: +86-159-2625-8658; Fax: +86-027-8728-2010

This file includes:

Supplementary Figure S1-S5

Supplementary Table S1-S7

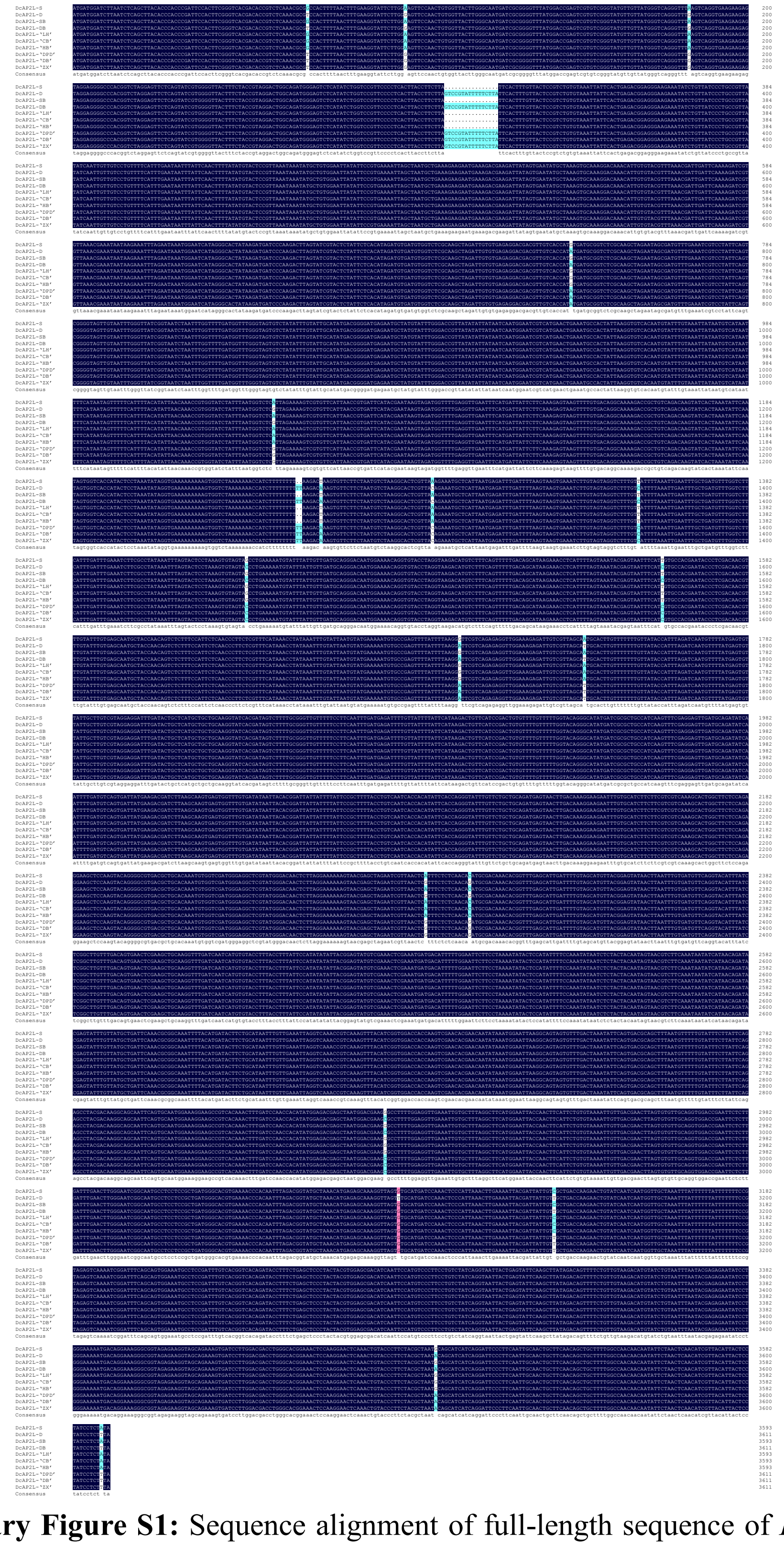

Supplementary Figure S1: Sequence alignment of full-length sequence of *DcAP2L*. *DcAP2L-S*, *DcAP2L-D*, *DcAP2L-SB*, *DcAP2L-DB*, *DcAP2L*-‘LH’, *DcAP2L*-‘CB’, *DcAP2L*-‘HB’, *DcAP2L*-‘DPD’, *DcAP2L*-‘DB’, and *DcAP2L*-‘ZX’ represent full-length sequence of *DcAP2L* in ‘MH’, ‘X4’, single flower bulk of F_2, double flower bulk of F_2, ‘LH’, ‘CB’, ‘HB’, ‘DPD’, ‘DB’, and ‘ZX’, respectively. Sequence differences are highlighted with light blue.

```
DcAP2L-S     ATGATGGATCTTAATCTCAGCTTACACCCACCCGATTCCACTTCGGGTCACGACACCGTCTCAAACGCGGCCACTTTTAACTTTGAAGGTATTCTTGGAA    100
DcAP2L-D     ATGATGGATCTTAATCTCAGCTTACACCCACCCGATTCCACTTCGGGTCACGACACCGTCTCAAACGCGTCCACTTTTAACTTTGAAGGTATTCTTGGGA    100
DcAP2L-SB    ATGATGGATCTTAATCTCAGCTTACACCCACCCGATTCCACTTCGGGTCACGACACCGTCTCAAACGCGGCCACTTTTAACTTTGAAGGTATTCTTGGAA    100
DcAP2L-DB    ATGATGGATCTTAATCTCAGCTTACACCCACCCGATTCCACTTCGGGTCACGACACCGTCTCAAACGCGTCCACTTTTAACTTTGAAGGTATTCTTGGGA    100
Consensus    atgatggatcttaatctcagcttacacccacccgattccacttcgggtcacgacaccgtctcaaacgcg ccacttttaactttgaaggtattcttgg a

DcAP2L-S     GTTCCAACTGTGGTTACTTGGGCAATGATCGCGGGGTTTATGGACCGAGTCGTGTCGGGTATGTTGTTATGGGTCAGGGTTTAAGTCAGGTGAAGAAGAG    200
DcAP2L-D     GTTCCAACTGTGGTTACTTGGGCAATGATCGCGGGGTTTATGGACCGAGTCGTGTCGGGTATGTTGTTATGGGTCAGGGTTTGAGTCAGGTGAAGAAGAG    200
DcAP2L-SB    GTTCCAACTGTGGTTACTTGGGCAATGATCGCGGGGTTTATGGACCGAGTCGTGTCGGGTATGTTGTTATGGGTCAGGGTTTAAGTCAGGTGAAGAAGAG    200
DcAP2L-DB    GTTCCAACTGTGGTTACTTGGGCAATGATCGCGGGGTTTATGGACCGAGTCGTGTCGGGTATGTTGTTATGGGTCAGGGTTTGAGTCAGGTGAAGAAGAG    200
Consensus    gttccaactgtggttacttgggcaatgatcgcggggtttatggaccgagtcgtgtcgggtatgttgttatgggtcagggttt agtcaggtgaagaagag

DcAP2L-S     TAGGAGGGGCCCACGGTCTAGGAGTTCTCAGTATCGTGGGGTTACTTTCTACCGTAGGACTGGCAGATGGGAGTCTCATATCTGGGACAATGGAAAACAG    300
DcAP2L-D     TAGGAGGGGCCCACGGTCTAGGAGTTCTCAGTATCGTGGGGTTACTTTCTACCGTAGGACTGGCAGATGGGAGTCTCATATCTGGGACAATGGAAAACAG    300
DcAP2L-SB    TAGGAGGGGCCCACGGTCTAGGAGTTCTCAGTATCGTGGGGTTACTTTCTACCGTAGGACTGGCAGATGGGAGTCTCATATCTGGGACAATGGAAAACAG    300
DcAP2L-DB    TAGGAGGGGCCCACGGTCTAGGAGTTCTCAGTATCGTGGGGTTACTTTCTACCGTAGGACTGGCAGATGGGAGTCTCATATCTGGGACAATGGAAAACAG    300
Consensus    taggaggggcccacggtctaggagttctcagtatcgtggggttactttctaccgtaggactggcagatgggagtctcatatctgggacaatggaaaacag

DcAP2L-S     GTGTACCTAGGAGGATTTGATACTGCTCATGCTGCTGCAAGGGCATATGATCGCGCTGCCATCAAGTTTCGAGGAGTTGATGCAGATATCAATTTTGATG    400
DcAP2L-D     GTGTACCTAGGAGGATTTGATACTGCTCATGCTGCTGCAAGGGCATATGATCGCGCTGCCATCAAGTTTCGAGGAGTTGATGCAGATATCAATTTTGATG    400
DcAP2L-SB    GTGTACCTAGGAGGATTTGATACTGCTCATGCTGCTGCAAGGGCATATGATCGCGCTGCCATCAAGTTTCGAGGAGTTGATGCAGATATCAATTTTGATG    400
DcAP2L-DB    GTGTACCTAGGAGGATTTGATACTGCTCATGCTGCTGCAAGGGCATATGATCGCGCTGCCATCAAGTTTCGAGGAGTTGATGCAGATATCAATTTTGATG    400
Consensus    gtgtacctaggaggatttgatactgctcatgctgctgcaagggcatatgatcgcgctgccatcaagtttcgaggagttgatgcagatatcaattttgatg

DcAP2L-S     TCAGTGATTATGAAGACGATCTTAAGCAAATGAGTAACTTGACAAAGGAAGAATTTGTGCATCTTCTTCGTCGTCAAAGCACTGGCTTCTCCAGAGGAAG    500
DcAP2L-D     TCAGTGATTATGAAGACGATCTTAAGCAAATGAGTAACTTGACAAAGGAAGAATTTGTGCATCTTCTTCGTCGTCAAAGCACTGGCTTCTCCAGAGGAAG    500
DcAP2L-SB    TCAGTGATTATGAAGACGATCTTAAGCAAATGAGTAACTTGACAAAGGAAGAATTTGTGCATCTTCTTCGTCGTCAAAGCACTGGCTTCTCCAGAGGAAG    500
DcAP2L-DB    TCAGTGATTATGAAGACGATCTTAAGCAAATGAGTAACTTGACAAAGGAAGAATTTGTGCATCTTCTTCGTCGTCAAAGCACTGGCTTCTCCAGAGGAAG    500
Consensus    tcagtgattatgaagacgatcttaagcaaatgagtaacttgacaaaggaagaatttgtgcatcttcttcgtcgtcaaagcactggcttctccagaggaag

DcAP2L-S     CTCCAAGTACAGGGGCGTGACGCTGCACAAATGTGGTCGATGGGAGGCTCGTATGGGACAACTCTTAGGAAAAAAGTACATTTATCTCGGCTTGTTTGAC    600
DcAP2L-D     CTCCAAGTACAGGGGCGTGACGCTGCACAAATGTGGTCGATGGGAGGCTCGTATGGGACAACTCTTAGGAAAAAAGTACATTTATCTCGGCTTGTTTGAC    600
DcAP2L-SB    CTCCAAGTACAGGGGCGTGACGCTGCACAAATGTGGTCGATGGGAGGCTCGTATGGGACAACTCTTAGGAAAAAAGTACATTTATCTCGGCTTGTTTGAC    600
DcAP2L-DB    CTCCAAGTACAGGGGCGTGACGCTGCACAAATGTGGTCGATGGGAGGCTCGTATGGGACAACTCTTAGGAAAAAAGTACATTTATCTCGGCTTGTTTGAC    600
Consensus    ctccaagtacaggggcgtgacgctgcacaaatgtggtcgatgggaggctcgtatgggacaactcttaggaaaaaagtacatttatctcggcttgtttgac

DcAP2L-S     AGTGAACTCGAAGCTGCAAGAGCCTACGACAAGGCAGCAATTCAGTGCAATGGAAAGGAAGCCGTCACAAACTTTGATCCAACCACATATGGAGACGAGC    700
DcAP2L-D     AGTGAACTCGAAGCTGCAAGAGCCTACGACAAGGCAGCAATTCAGTGCAATGGAAAGGAAGCCGTCACAAACTTTGATCCAACCACATATGGAGACGAGC    700
DcAP2L-SB    AGTGAACTCGAAGCTGCAAGAGCCTACGACAAGGCAGCAATTCAGTGCAATGGAAAGGAAGCCGTCACAAACTTTGATCCAACCACATATGGAGACGAGC    700
DcAP2L-DB    AGTGAACTCGAAGCTGCAAGAGCCTACGACAAGGCAGCAATTCAGTGCAATGGAAAGGAAGCCGTCACAAACTTTGATCCAACCACATATGGAGACGAGC    700
Consensus    agtgaactcgaagctgcaagagcctacgacaaggcagcaattcagtgcaatggaaaggaagccgtcacaaactttgatccaaccacatatggagacgagc

DcAP2L-S     TAATGGACGAAGGGCCTTTTGGAGGTGGACCGAATTCTCTTGATTTGAACTTGGGAATCGGCAATGCCTCCTCCGCTGATGGGCACGTGAAAACCCACAA    800
DcAP2L-D     TAATGGACGAAGCGCCTTTTGGAGGTGGACCGAATTCTCTTGATTTGAACTTGGGAATCGGCAATGCCTCCTCCGCTGATGGGCACGTGAAAACCCACAA    800
DcAP2L-SB    TAATGGACGAAGGGCCTTTTGGAGGTGGACCGAATTCTCTTGATTTGAACTTGGGAATCGGCAATGCCTCCTCCGCTGATGGGCACGTGAAAACCCACAA    800
DcAP2L-DB    TAATGGACGAAGCGCCTTTTGGAGGTGGACCGAATTCTCTTGATTTGAACTTGGGAATCGGCAATGCCTCCTCCGCTGATGGGCACGTGAAAACCCACAA    800
Consensus    taatggacgaag gccttttggaggtggaccgaattctcttgatttgaacttgggaatcggcaatgcctcctccgctgatgggcacgtgaaaacccacaa

DcAP2L-S     TTTAGACGGTATGCTAAACATGAGAGCAAAGAGTCAAAATCGGATTTCAGCAGTGGAAATGCCTCCGATTTGTCACGGTCACAGATACCTTTCTGAGCTC    900
DcAP2L-D     TTTAGACGGTATGCTAAACATGAGAGCAAAGAGTCAAAATCGGATTTCAGCAGTGGAAATGCCTCCGATTTGTCACGGTCACAGATACCTTTCTGAGCTC    900
DcAP2L-SB    TTTAGACGGTATGCTAAACATGAGAGCAAAGAGTCAAAATCGGATTTCAGCAGTGGAAATGCCTCCGATTTGTCACGGTCACAGATACCTTTCTGAGCTC    900
DcAP2L-DB    TTTAGACGGTATGCTAAACATGAGAGCAAAGAGTCAAAATCGGATTTCAGCAGTGGAAATGCCTCCGATTTGTCACGGTCACAGATACCTTTCTGAGCTC    900
Consensus    tttagacggtatgctaaacatgagagcaaagagtcaaaatcggatttcagcagtggaaatgcctccgatttgtcacggtcacagatacctttctgagctc

DcAP2L-S     CCTACTACGTGGAGCGACATCAATTCCATGTCCCTTCCTGTCTATCAGGAAAGGGCGGTAGAGAAGGTAGCAGAAAGTGATCCTTGGACGACCTGGGCAC   1000
DcAP2L-D     CCTACTACGTGGAGCGACATCAATTCCATGTCCCTTCCTGTCTATCAGGAAAGGGCGGTAGAGAAGGTAGCAGAAAGTGATCCTTGGACGACCTGGGCAC   1000
DcAP2L-SB    CCTACTACGTGGAGCGACATCAATTCCATGTCCCTTCCTGTCTATCAGGAAAGGGCGGTAGAGAAGGTAGCAGAAAGTGATCCTTGGACGACCTGGGCAC   1000
DcAP2L-DB    CCTACTACGTGGAGCGACATCAATTCCATGTCCCTTCCTGTCTATCAGGAAAGGGCGGTAGAGAAGGTAGCAGAAAGTGATCCTTGGACGACCTGGGCAC   1000
Consensus    cctactacgtggagcgacatcaattccatgtcccttcctgtctatcaggaaagggcggtagagaaggtagcagaaagtgatccttggacgacctgggcac

DcAP2L-S     GGAAACTCCAAGGAACTCAAACTGTACCCTTCTACGCTAATGCAGCATCATCAGGATTCCCTTCAATTGCAACTGCTTCAACAGCTGCTTTTGGCCAACA   1100
DcAP2L-D     GGAAACTCCAAGGAACTCAAACTGTACCCTTCTACGCTAATACAGCATCATCAGGATTCCCTTCAATTGCAACTGCTTCAACAGCTGCTTTTGGCCAACA   1100
DcAP2L-SB    GGAAACTCCAAGGAACTCAAACTGTACCCTTCTACGCTAATGCAGCATCATCAGGATTCCCTTCAATTGCAACTGCTTCAACAGCTGCTTTTGGCCAACA   1100
DcAP2L-DB    GGAAACTCCAAGGAACTCAAACTGTACCCTTCTACGCTAATACAGCATCATCAGGATTCCCTTCAATTGCAACTGCTTCAACAGCTGCTTTTGGCCAACA   1100
Consensus    ggaaactccaaggaactcaaactgtacccttctacgctaat cagcatcatcaggattcccttcaattgcaactgcttcaacagctgcttttggccaaca

DcAP2L-S     ACAATATTCTAACTCAACATCGTTACATTACTCCTATCCTCTATA                                                           1145
DcAP2L-D     ACAATATTCTAACTCAACATCGTTACATTACTCCTATCCTCTTTA                                                           1145
DcAP2L-SB    ACAATATTCTAACTCAACATCGTTACATTACTCCTATCCTCTATA                                                           1145
DcAP2L-DB    ACAATATTCTAACTCAACATCGTTACATTACTCCTATCCTCTTTA                                                           1145
Consensus    acaatattctaactcaacatcgttacattactcctatcctct ta
```

Supplementary Figure S2: Sequence alignment of CDS sequence of *DcAP2L*. *DcAP2L-S*, *DcAP2L-D*, *DcAP2L-SB* and *DcAP2L-SB* represent CDS sequence of *DcAP2L* in 'MH', 'X4', single flower bulk and double flower bulk of F_2, respectively. Sequence differences are highlighted with light blue.

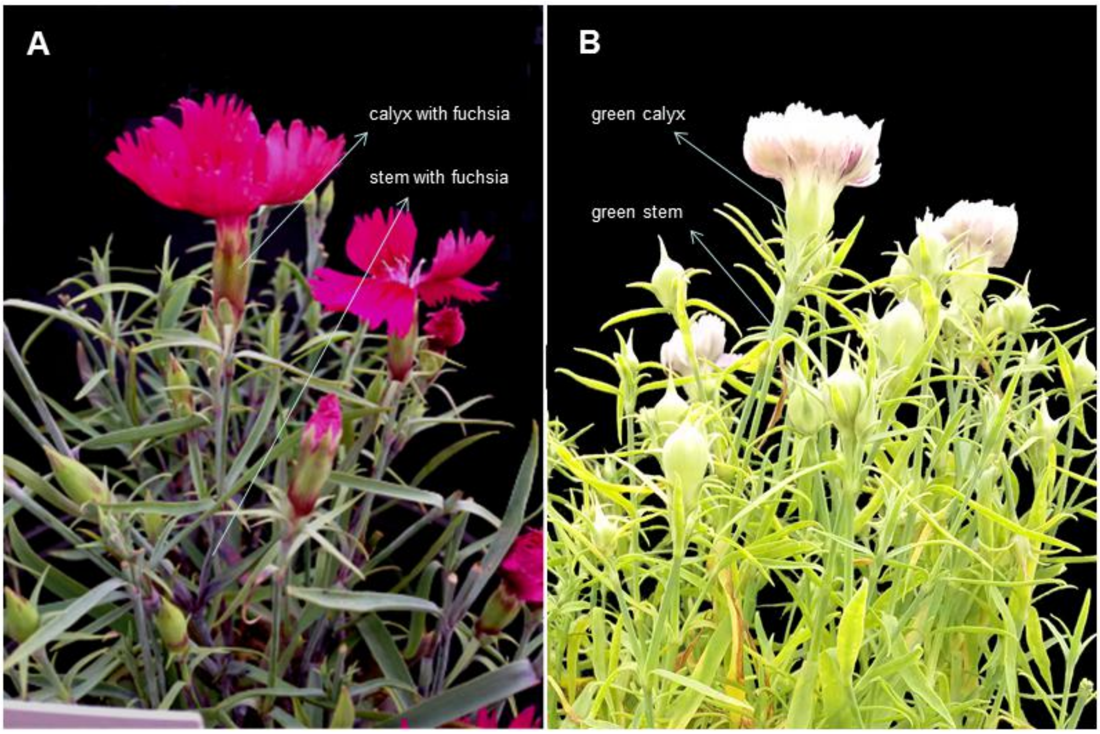

Supplementary Figure S3: Flower and stem color characteristics of the two parents.
A, ‘MH’ shows calyxes and stem with fuchsia.
B, ‘X4’ shows green calyxes and stem which are different from ‘MH’.

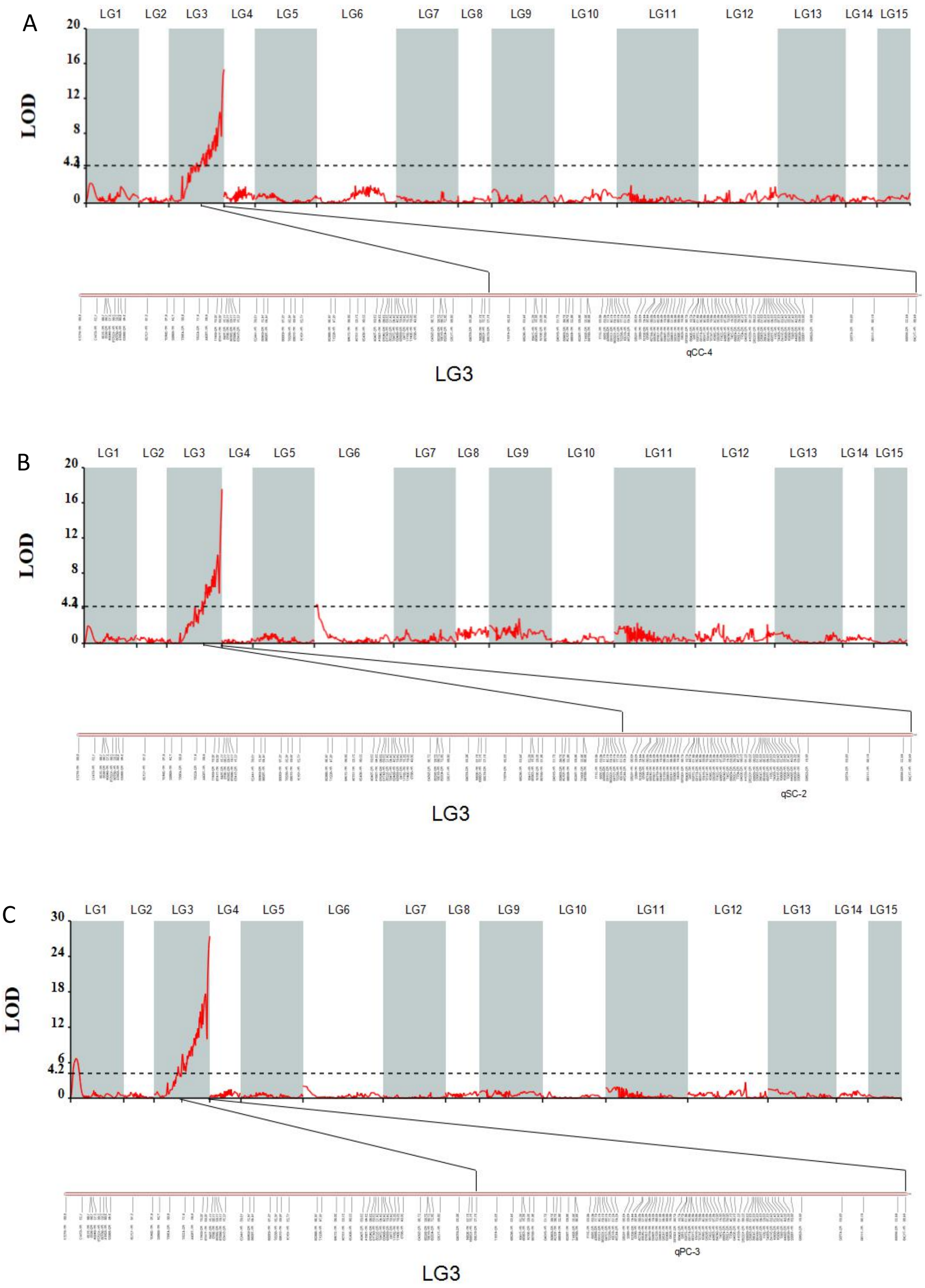

Supplementary Figure S4: Genetic location of QTLs for color related trait. A, B and C indicate genetic location of QTLs for calyxes color, stem color and style color of pistil, respectively. The horizontal dashed line represents a linkage group-wise logarithm of odds (LOD) significance threshold of 4.3 for calyxes color trait, and 4.2 for stem color and style color of pistil trait. qCC-4, qSC-2 and qPC-3 are most significant QTL regions for three color-related traits with the same peak position correspond the highest LOD value.

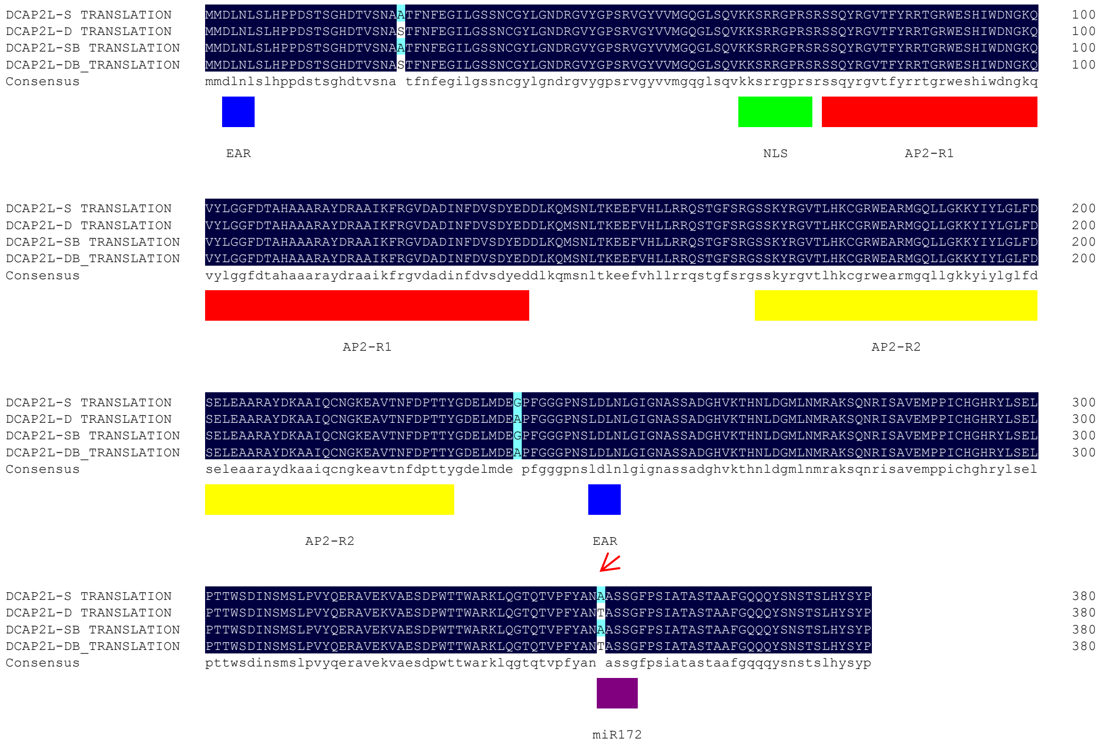

Supplementary Figure S5: Sequence alignment of putative amino acid sequence of *DcAP2L*. *DcAP2L-S_translation*, *DcAP2L-D_translation*, *DcAP2L-SB_translation* and *DcAP2L-DB_translation* represent putative amino acid sequence of *DcAP2L* in ‘MH’, ‘X4’ and single flower and double flower bulk of F_2 population, respectively. The missense mutation that lies within the miR172 target site of *DcAP2* is marked by red arrow. Color boxes beneath the sequence indicate the position of the two AP2 domains (AP2-R1 and AP2-R2, in red and yellow respectively), of the EAR motif-like sequences (in dark blue), of the nuclear localization signal (NLS, in green), and the sequence derive from miR172 target site (in purple).

Supplementary Table S1: Evaluation criteria for agronomic traits and assignment for qualitative characters of *D.Chinensis*.

Trait	Abbreviation	Evaluation criterion	Assignment
Double flower trait (petal number)	DFT	Average petal number of nine flowers in each plant	/
Stamen number	SN	Average stamen number of nine flowers in each plant	/
Stem color	SC	The stem color with green and fuchsia (part or all with fuchsia)	1 represent stem color with green; 2 represent stem color with fuchsia
Branch number	BN	Number of branches on main stem	/
Leaf with wax	LWW	It's actually a white appendage on the leaf, we wiped the leaves with a black cloth to judge whether the leaf have this white appendage	1 represent the leaf have this white appendage; 2 represent the leaf have no this white appendage
Leaf width	LW	Average width and length of two leaves which located at the fifth pair from the top down	/
Leaf length	LL		/
Plant height	PH	One plant measured three times	/
Plant width	PW		/
Stem diameter	SD	Measured three times for the stem diameter of main stem which next to the soil	/
Calyx color	SC	The calyx color with green and fuchsia (part or all with fuchsia)	1 represent calyx color with green; 2 represent calyx color with fuchsia
Style color of the pistil	PC	The style color with white and fuchsia(part or all with fuchsia)	1 represent style color with white; 2 represent style color with fuchsia

Supplementary Table S2: Primers used for quantitative PCR and cloning.

Name	Application	Forward primer (5'-3')	Reverse primer (5'-3')
DcAP2L	qRT-PCR	GATGGGAGGCTCGTATGG	CCGTCTAAATTGTGGGTTTT
DcAGa	qRT-PCR	GCATTAGTAGAATCCGATCCAA	TCGTAGTCACCACCACCCG
DcAGb	qRT-PCR	CGAATCCGATCCAAAAAGAATG	TCGTACTCGCTGCTACCTCC
DcGAPDH	qRT-PCR	CGGAAAGTTGACTGGTATGGC	CATCCTCGGTGTAGCCCAAAAT
DcAP2L	cloning	TGTATTCCCCTTTGTACACTCAC	GACACGATTCAGACTATGAACACT
miR172a-precursor	cloning	TGTGGTCTCAAATGTGCTGTGGCATCATCAAGATTCAC	TGTGGTCTCAAAGCGCCGATGCAGCATCATCAAGA

.

Supplementary Table S3: Number of floral organs of ‘MH’, ‘X4’and F_1 plants in *D. chinensis*.

Floral organ	‘MH’	‘X4’	F_1
Sepal	5.00±0.00	5.00±0.00	5.00±0.00
Petal	5.00±0.00	25.43±3.18	9.52±1.65
Chimera petal-stamen	0.00±0.00	3.43±1.93	2.34±1.58
Stamen	10.00±0.00	13.62±2.26	8.54±2.33
Style	2.00±0.00	2.60±0.69	2.00±0.00
Total	22.00±0.00	50.08±4.95	27.4±2.22

Nine flowers in each plant and six plants in each material are measured.

Deviation values represents standard deviation.

Supplementary Table S4: Summary of sequence data from parents and 140 F_2 progenies.

Sample ID	Raw reads	Clean reads	GC rate(%)	Q30 rate(%)	Average Depth
‘X4’	5223010	5222674	41.27	92.59	24.22
‘MH’	5287378	5287064	40.96	92.74	24.92
Average of F_2	2652103	2651894	41.44	90.05	9.60

Supplementary Table S5: Type of variation of the identified 2353 SNP markers for constructing the genetic linkage map.

Type of variation	NO. of SNP	Rate（%）
A/C	230	9.77
C/G	164	6.97
G/A	686	29.15
T/A	324	13.77
C/T	747	31.75
G/T	202	8.59
Total	2353	100

Supplementary Table S6: Significance test of agronomic characters between parents and character statistics of parents and offspring.

Trait	Generation			
	'MH'	'X4'	F_1	F_2
Double flower trait (petal number)**	5.00±0.00	25.43±3.18	9.52±1.65	11.89±6.21
Stamen number	10.00±0.00	13.62±2.26	8.54±2.33	9.51±1.89
Stem color**	2.00±0.00	1.00±0.00	1.00±0.00	1.54±0.50
Branch number*	12.25±2.38	10.25±1.64	19.25±1.48	12.26±3.78
Leaf with wax**	1.00±0.00	2.00±0.00	1.00±0.00	1.18±0.38
Leaf width(mm)	6.82±0.84	6.20±0.72	8.47±0.53	7.21±1.83
Leaf length(cm)	7.32±0.56	6.05±0.58	8.03±0.90	6.55±1.06
Leaf length(cm)	16.75±0.80	17.27±1.46	36.02±3.22	22.74±5.61
Plant width**	21.77±3.05	14.77±2.05	25.67±1.41	22.81±4.71
Stem diameter	2.74±0.46	2.23±0.31	3.61±0.22	3.03±0.71
Calyx color**	2.00±0.00	1.00±0.00	1.00±0.00	1.54±0.50
Style color of the pistil**	1.00±0.00	2.00±0.00	2.00±0.00	1.36±0.48

Note: DPS7.05 software is used for character statistics, and *t*-test is used to test significant difference of agronomic characters between two parents. **indicates $P< 0.01$;*indicates $P< 0.05$. Deviation value represents standard deviation.

Supplementary Table S7: The sequence of fragment containing the sequence of pre-miR172a of *D.chinensis*.

>seq-pre-miR172a

GGTAAGGCTTTTCTTTCAAGATTCTCTCTGCCGACAGTGGTCCCAAAGATGGACCCCC
ACCCACGAGGAGCATCGTGGAAAAAGAAGAGGTTCCAACCACGTCTACAAAGCAAGT
GGATTGATGTGACATCTCCACTGACGTAAGGGATGACGCACAATCCCACTATCCTTCGC
AAGACCCTTCCTCTATATAAGGAAGTTCATTTCATTTGGAGAGGACACGCTCGAGTATA
AGAGCTCATTTTTACAACAATTACCAACAACAACAAACAACAAACAACATTACAATTA
CATTTACAATTATCGATACAATGTGCTGTGGCATCATCAAGATTCACATGCCTCACTTCC
ATTGACCTTTCTTTCCCACTTATTTTTTTGTTATTTTGTTAAAAAAAATTGTTTTTTTTTTT
TTTTAATATTATTAGATGTGAAGCTTTCATATATGTGGTCCTTCAAATAATTGTATCAGAA
AATATTATTGTGTTTTTATTATTATTATTGGGTCCATTTTATTTTTATTTTTGTGGGAAGAG
GTCAAGAGGAAAACAAATGATATGAGAATCTTGATGATGCTGCATCGGCGCTTGTCCTG
CTTTAATGAGATATGCGAGAAGCCTATGATCGCATGATATTTGCTTTCAATTCTGTTGTG
CACGTTGTAAAAAACCTGAGCATGTGTAGCTCAGATCCTTACCGCCGGTTTCGGTTCAT
TCTAATGAATATATCACCCGTTACTATCGTATTTTTATGAATAATATTCTCCGTTCAATTTA
CTGATTGTACCCTACTACTTATATGTACAATATTAAAATGAAAACAATATATTGTGCTGAA
TAGGTTTATAGCGACATCTATGATAGAGCGCCACAATAACAAACAATTGCGTTTTATTAT
TACAAATCCAATTTTAAAAAAAGCGGCAGAACCGGTCAAACCTAAAAGACTGATTACA
TAAATCTTATTCAAATTTCAAAAGTGCCCCAGGGGCTAGTATCTA
